# The assessment of the quality of campus public spaces as key parts of the learning landscape: experience from a crowdsensing study on the Third Campus of Jagiellonian University, Krakow, Poland

**DOI:** 10.1057/s41289-023-00224-1

**Published:** 2023-06-03

**Authors:** Jarosław Działek, Bartłomiej Homiński, Magdalena Miśkowiec, Agnieszka Świgost-Kapocsi, Krzysztof Gwosdz

**Affiliations:** 1 Institute of Geography and Spatial Management, Faculty of Geography and Geology, Jagiellonian University, ul. Gronostajowa 7, 30-387 Kraków, Poland; 2 Chair of Urbanism and Architecture of City Structure, Faculty of Architecture, Cracow University of Technology, ul. Podchorążych 1, 30-084 Kraków, Poland

**Keywords:** Learning landscape, University spaces, Campus, Public spaces, Crowdsensing

## Abstract

The learning landscape concept reflects shifts in the methods of learning and conducting research in universities. Public spaces within university campuses should constitute an essential component of the learning landscape as arenas of planned and serendipitous encounters, which may foster creativity and trans-disciplinary networking. However, their spatial configurations remain an under-researched topic. This paper assesses the quality of public spaces on campus based on the results of a crowdsensing survey. The Third Campus of Jagiellonian University in Krakow was selected as a case study; this is one of the largest projects of this kind carried out in Poland since the political and economic transformation of 1989. The behaviour of users provides evidence of the generally low quality of the public spaces despite the advantages of the urban layout of the campus. The paper proposes recommendations that may bring the spatial organisation of the campus closer to a fully fledged learning landscape.

## Introduction

The concept of the learning landscape (or knowledgescape) refers to the changing context of the functioning of university spaces, which reflects recent shifts in research and education (Dugdale 2009; Winnicka-Jasłowska 2012; Backman et al. 2019; Cox et al. 2020; Soares et al. 2020a). These shifts involve the emergence of various learning and research tasks and their settings that combine individual and collaborative efforts, formal and informal knowledge exchange, self-education and interactive co-learning and co-creation with the use of both digital and physical resources and means (Nordquist et al. 2013; Asher et al. 2017; Whyte 2018; Backman et al. 2019; Cox et al. 2020). In this context, the spaces of learning and research go beyond typical closed classrooms and laboratories. These include outdoor areas of intensive socialising, both planned and serendipitous, that favour the sharing of ideas and transdisciplinary cross-fertilisation. Furthermore, these areas also function as spaces that allow one to recover from mental fatigue and restore psychological well-being (Griffith 1994; Biddulph 1999; Aydin and Ter 2008; Matsuoka and Kaplan 2008; McFarland et al. 2008; Thody 2011; Speake et al. 2013; Seitz et al. 2014; Lau et al. 2014; Liprini and Coetzee 2017; Göçer et al. 2018; Cox et al. 2020; Sikorski et al. 2020; Soares et al. 2020a; Yaylali-Yildiz et al. 2022).

Thus, higher education institutions need to redefine existing configurations of university and campus spaces of learning and discovery and adapt them to respond to the changing expectations of students and researchers (Biddulph 1999; Dugdale 2009; O’Rourke and Baldwin 2016; Özkan et al. 2017; Schwenius et al. 2017; Göçer et al. 2018; Cox et al. 2020; Sikorski et al. 2020). Moreover, the concept of learning landscape calls for more connections between university and campus spaces and the urban fabric around to create a ‘univer-city’ (Maurrasse 2001; Jones 2017; den Heijer and Curvelo Magdaniel 2018; Yu et al. 2018; Čibik and Štěpánková 2020).

These considerations are related to a broader debate on the role of public spaces in enhancing social interactions and, consequently, stimulating creativity and the co-creation of knowledge (Costa and Lopes 2015; Carmona 2019). Campus researchers (Özkan et al. 2017; Göçer et al. 2018; Ozbil et al. 2018; Čibik and Štěpánková 2020; Soares et al. 2020b) refer to the concepts of the Danish urban planner Gehl (2008), who postulates the creation of high-quality spaces that enable the pursuit of not only the necessary activities (e.g. moving between buildings, or from a building to a bus stop), but also optional activities (e.g. strolling, stopping, sitting down, watching). He posits that the physical layout of public spaces is one of important factors supporting their liveability. More than to the aesthetic qualities, it refers to features such as scale and proportions, psychological considerations including safety, sensory experiences, physical comfort, diversity of functions (Gehl et al. 2006). Successful public spaces offer individuals and groups the opportunity to walk, sit, relax, observe, converse, and listen according to their specific needs. Consequently, they attract a higher number of diverse groups of users involved in various forms of activities and longer duration of their stays (Gehl 1986, 2007; see Carmona 2019). Similarly at university campuses “people want to gather, socialise, study and be creative in a comfortable and inviting space that offers shelter and shade from the elements, places to sit, eat and drink, and a space in which to enjoy cultural and artistic activities” (O’Rourke and Baldwin 2016, p. 114). Therefore, when contemporary campuses are planned, they should be thought of as complexes of buildings on a human scale, designed with pedestrians in mind, with places and functions enabling interpersonal contacts and social participation—places that connect the university with its surroundings (Domae 2017; Schwenius et al. 2017)). Properly designed common spaces inside buildings and public spaces between them (Abu-Ghazzeh 1999; Arefi and Triantafillou 2005; Salama 2008; Özkan et al. 2017; Sikorski et al. 2020) could be considered a key element in the practical implementation of the learning landscape concept.

In this context, there is a noticeable shortage of studies on campus public spaces (Speake et al. 2013; Schwenius et al. 2017; Cox et al. 2020; Sikorski et al. 2020; Soares et al. 2020a, 2020b), despite “their critical role in learning and community life” (Göçer et al. 2018, p. 126). If universities want to focus on implementing the idea of the learning landscape, they should thoroughly examine whether their public spaces meet the needs and expectations of their academic and non-academic users, and what could be done to strengthen their role in supporting the exchange of knowledge and skills between them.

This is especially important in the context of Poland (and other Central and Eastern European countries), where the processes of creating new university campuses intensified in the first two decades of the twenty-first century, with projects usually imitating past, modernist models of specialised, isolated suburban campuses (Kapecki 2015; Żabicki 2016). The construction projects focused on erecting buildings to satisfy the space needs of individual faculties or units—a consequence of siloed thinking at universities (see Nordquist et al. 2013; Winnicka-Jasłowska 2015). Meanwhile, open spaces, mixed-use buildings, and areas for socialising within the buildings, the purpose of which would be to integrate members of the academic and non-academic communities, were rarely created.

The aim of this paper is thus to assess the quality of public spaces within one of the new flagship university campuses in Poland in the context of the idea of the learning landscape, using the technique of mobile crowdsensing. We want to find out how people use the public spaces on the Third Campus of Jagiellonian University, understand why some of them are underused, and suggest how they can be improved. The crowdsensing survey results are then expertly assessed by the authors (from inside and outside the campus), who represent human geography, architecture and urban planning, and activists working to improve the situation on the campus. Our findings contribute to a better understanding of the functioning of campus spaces and serve as a basis for recommendations on how to design and redesign them.

## Research design

Studies of public spaces within university campuses are relatively rare, but they employ a number of research methods used to assess public spaces in cities in general. Among them, two groups of approaches prevail: those relying on expert assessments based on field observations, behavioural mapping and photographic documentation; those whereby user opinions are obtained through questionnaire surveys, in-depth interviews, research walks, and visual methods (see review of research methods in Göçer et al. 2018).

In traditional surveys relating to public spaces, the respondents are typically not present in the places they assess. In this study, we employed an approach based on a mobile application (known as mobile crowdsensing or mobile participatory sensing) that allowed respondents to complete the questionnaire at different locations within the campus itself (Kanhere 2013; Aanensen et al. 2014; Kim et al. 2019). Crowdsensing involves generating a large amount of field data, often by members of communities connected with an area. They can share local knowledge, which can then be used in decision-making. Crowdsensing in spatial research enables more data to be collected from active participants at a lower cost. However, there may be difficulties associated with the recruitment of participants, the non-inclusion of certain social groups, technical limitations, incompleteness of data due to the withdrawal of participants during the survey, and occasionally, with data distortions due to the malicious behaviour of some participants (Burke et al. 2006; Kanhere 2013).

The study used the Epicollect5 platform, provided by Imperial College London, which can be used for the preparation, collection and processing of crowdsensing field surveys. One advantage is that it can not only be used for collecting text responses, but also for obtaining geolocated images, audio and video recordings (Aanensen et al. 2014). The Epicollect5 platform was originally intended to improve the collection of epidemiological data, but since then it was also used in spatial analyses (Bryant et al. 2013; France et al. 2015; Ahmed et al. 2019). To our knowledge, mobile crowdsensing, especially using the Epicollect5 platform, has not previously been used for campus public spaces assessment. It is expected, however, that people-centric mobile crowdsensing platforms with both user- and sensor-generated data will more and more often be utilised to support urban design (Xiang et al. 2017).

The participants in the study were first-year students of the Institute of Geography and Spatial Management of the Jagiellonian University who had spent eight months studying on the campus. During field surveys, they were asked to evaluate outdoor locations of their choice on the campus that are available to campus users without restrictions. They moved individually at times convenient to them, completing a short questionnaire at each location. The questionnaire included the following: (1) assessment of comfort whether walking or stationary from the perspective of a pedestrian; (2) their own experience of using a given location; (3) the behaviour of other people that they observed; (4) elements they liked or disliked (with the option of taking pictures). In total, sixty students participated in the survey which was conducted in the first half of June 2018 on days with warm (max temperature of 21 to 27 °C), sunny or slightly cloudy weather. They assessed 960 locations, of which 953 were included in the final analysis, with those observations having an accuracy of location identification below the accepted range being discarded.

The paper is based, for the most part, on the observations of other campus users made by the survey participants. For each location and its surroundings, we asked participants to observe whether there was anyone within a given space. If so, they noted whether that was a single person (up to 2–3 persons in total), several people (4–9) or a larger number of people (more than 10). Following this, they marked the predominant type of behaviour observed, i.e. whether the users of the space mainly walked without stopping, or whether they were chiefly sitting, standing, talking or strolling leisurely, or, eventually, that both activities were noted in similar proportions.

In the interpretation of the results, we assumed that the behaviour of people in space that is observed indirectly reflects the attractiveness of a given space for pedestrians. As proposed by Gehl (2008), this postulates that while in a ‘bad’ space, which is unattractive in terms of its physical layout, people will mainly perform necessary activities, i.e. they will walk without stopping, while in a ‘good’ space they will tend to perform optional activities and socialise—stop, sit down, talk, and establish and maintain social contacts. The analysis of the photographs taken by the participants together with their comments has provided an insight into why they may consider the individual public spaces to be more or less attractive. The results were confronted with the expert knowledge of the authors. However, the limitation of our approach is that we were not able to identify different groups of users, that may have different preferences. We understand this study as the first step before undertaking more in-depth quantitative and qualitative studies of these public spaces.

The unevenly distributed locations with the respondents’ answers were aggregated using a regular grid with points spaced every 12.5 m, around which circles with a radius of 12.5 m were drawn. For the locations that found themselves within the individual circles, we calculated the mean values on the basis of the respondents’ answers. Subsequently, we interpolated some of the data to be presented as smoothed areas that would facilitate the interpretation of the collected data.

**Fig. 1:**
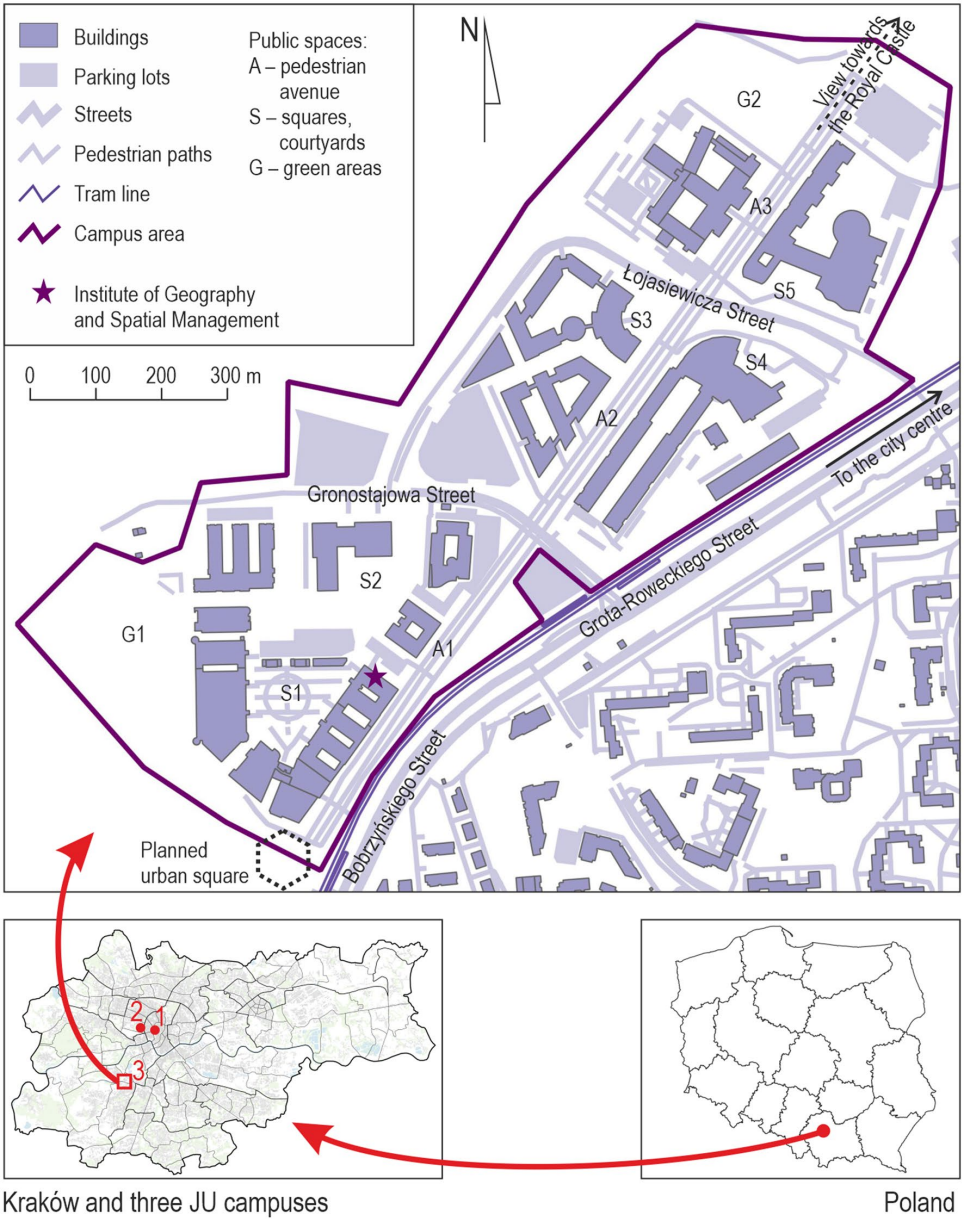
The spatial structure of the Jagiellonian University Third Campus with the main public spaces. *Source* Own study

During the analysis, we paid particular attention to the areas which had the greatest potential to serve as public spaces, and which could play an important role in creating a learning landscape within the campus (Fig. 1). These include the central pedestrian avenue of the campus, which should constitute its backbone, divided by streets into three sections—southern (A1), middle (A2) and northern (A3). Additionally, the key spaces comprise five squares and courtyards—two internal ones (S1 and S2) and three in front of the main entrances to the faculty buildings (S3, S4 and S5)—and two green areas—in the south (G1) and in the north (G2).

## Research area: the Third Campus within the spatial structure of the university and the city

The study examines the Campus of the 600th Anniversary of the Jagiellonian University Revival (in Polish Kampus 600-lecia Odnowienia Uniwersytetu Jagiellońskiego) in Krakow, also known as the Third Campus. Jagiellonian University, founded in 1364, is the oldest university in Poland and the second oldest institution of higher education in Central Europe. Its earliest buildings, which date back to the fifteenth century, are located within the multifunctional urban fabric of the UNESCO-listed medieval city, which is an outstanding example of an architectural ensemble of exceptional value in terms of both its townscape and its individual monuments (*Historic Centre of Kraków*…). The second campus of Jagiellonian University was built in the 1960s in the west side of the Old Town. The buildings of Jagiellonian University and other schools of higher education erected at that time complement the inter-war urban layout of a grand avenue lined with monumental buildings for culture and education, which goes around the Old Town to the west. Although the free-standing university buildings of the 1960s surrounded by greenery are a manifestation of the modernist urban idea of a ‘university district’, with the underlying principle of functional segregation, in practice, they take advantage of the proximity of the Old Town, which is only a few hundred metres away and with which they are functionally integrated. This district has a fairly well-developed infrastructure for students, with dormitories, student clubs, sports centres, etc.

The Third Campus is the newest and largest group of buildings of the Jagiellonian University in Kraków. It was built from 1998 to 2017 on a greenfield site in the south-western part of the city, about 4 km away from the Old Town (Jędrychowski 2007; Böhm 2008; Franaszek 2020). Beside the university premises, a vast area of over 135 hectares is occupied by technology parks and office buildings for IT and outsourcing companies. However, the science- and business-oriented sections are clustered into two, quite distant and unconnected parts of the area. There are ten buildings within the area erected by the Jagiellonian University which house institutes and faculties, of mainly natural sciences. There are also plans to build sports facilities and student dormitories. Of all the stages of the development of Jagiellonian University, the construction of the Third Campus has left the most distinct mark on the shape of Krakow, catalysing its development towards the south-west and enriching its space with a distinctive urban layout.

The development of the Third Campus is a compromise of the contradictory aspirations envisaged by the pre-existing general zoning plan, namely the protection of open natural landscape and the provision of high-intensity development envisaged for the future campus (Böhm 2008). Before the construction started, a conceptual framework for the development of the land was prepared for the area in the form of a coordination plan, which was based on an earlier landscape study. The study identified valuable buildings and their ensembles visible on the horizon. The key public spaces on the campus and in the technology park (streets, pedestrian avenues and squares) were laid out to correspond to the directions of views opening towards the above-mentioned historic features. The urban blocks delineated by these public spaces were designated for development. The pedestrian avenue (A1-A2-A3) that originates from the planned square at the south-west end of the campus along the view towards the Royal Castle became the central axis of the layout (Fig. 1). The avenue is nearly 1 km long and about 40 m wide (between the frontages), a pedestrian path with a double row of low trees. It symbolically connects the university area with the Old Town.

The above design has resulted in one of the most expressive spatial creations in Krakow after 1989. Its essence lies in the axial configuration of urban interiors, which evokes one of the most characteristic elements of Krakow’s urban layout, namely compositional axes terminated with significant buildings and monuments (Motak 2018). However, the legibility and imageability of the pedestrian avenue are undermined by the lack of distinctive terminations of the avenue within the campus itself (see Lynch 1960; Cullen 1971).

The plot areas delineated by the course of streets, walkways and other public spaces were assigned to the individual faculties. In the southern part of the area, there is an extensive biological sciences complex, which consists of two wings and a connecting hub with a lecture theatre, library and cafeteria. The hub was to serve as the southern entrance to the campus. This has not been achieved because the urban square from which this entrance was supposed to lead and which was envisaged in the coordination plan has not yet been built. Given the absence of the square, it is not possible to fully benefit from the potential of the cafe located there and the terrace adjacent to it, which were both to face the square. This complex, together with five smaller buildings, forms a compact arrangement centred around an irregular courtyard (S1 and S2), which was to form a ‘viewing plateau with a garden’ (Fikus 2002). The entrances to the buildings face onto it, which means that the yard could potentially act as the local centre of this section of the campus area. Its disadvantages, though, are a poor connection with the avenue (A1) across a car park and manoeuvring roads and being divided by utility buildings.

Four more faculty buildings, each with their own libraries, lecture theatres and cafeterias, were built alongside the central and northern sections of the avenue (A2 and A3), which reduces the opportunities for random interaction between campus users from its different parts. Some of the intentions of the coordination plan (later the zoning plan) can be clearly seen in the way the buildings are formed, as is manifested, inter alia, by the consistent setback line, as well as by the accentuation of the corners of the buildings. Nevertheless, the façades facing the avenue and other public spaces are usually long and passive with no accompanying programmes and little architectural detail to stimulate the curiosity of the users (see Gehl et al. 2006). Most of the buildings face the avenue with only side entrances which limits the flow of users along the avenue, and their random interactions (see Sikorski et al. 2020). The impression of monotony is enhanced by two strings of several dozen identical benches, facing each other alongside the avenue, looking out onto the windowless facades of buildings or even unattractive noise barriers (see Whyte 2018). An attractive view is only ensured in the vicinity of the S3 square thanks to a fountain and the old trees preserved here.

The campus is complemented by two green areas: one at the northern (G2) and one at the southern (G1) ends of the university area. The pedestrian avenue culminates with the first green area. Its attractiveness is largely due to the presence of old trees from before the construction of the campus and on differences in terrain elevation, which gives it an intimate character and allows users to enjoy a diversity of vistas. Both areas offer outdoor fitness features and equipment. The second green area is more problematic in terms of its role within the campus’s network of public spaces as it is cut off from the avenue (A1) and the adjacent courtyards (S1 and S2). However, it is eagerly frequented by residents of neighbouring estates. The planners intended it to be, together with the urban square yet to be completed, a kind of internal ‘seam’ or ‘stitch’ in the open area that connects the campus and the technology park. However, due to it lacking appropriate access for pedestrians from the campus side, this space does not add to integrating students and researchers with the local community.

The campus is connected with the centre of Krakow by a tram line built in 2012. There are three pairs of tram stops along the campus. The shortest and most frequented walking routes from the stops lead across the car parks and delivery areas to the secondary entrances of the university buildings, and the accompanying spatial setting is far from what could be considered to be the main entrance to the campus. Since the direction of the avenue and the flow of the pedestrians between the buildings and the tram stops do not overlap, the potential of the main campus axis is not fully exploited.

The existing interconnections between the campus and the neighbouring areas are relatively weak, both with the natural landscape park to the north and with the housing estate to the south. It is separated from the latter by wide, busy dual carriageway connecting the motorway bypass with the city centre. The street with the tram track is as much as fifty metres wide, which is emphasised by the presence of tall noise barriers. As a consequence, the street forms an onerous urban barrier, which hinders the functional and spatial connections between the campus and the neighbouring housing estates. The poor connections with the neighbouring areas mean that the city-forming potential of this public investment, which should be a role model in terms of urban planning, has not been properly exploited.

In terms of the quality of architecture, most of the buildings are assessed by university architecture researchers as representing an average level (Kapecki 2015). Despite the enormous organisational and financial effort, the campus lacks examples of outstanding architecture and public spaces, which are seen nowadays as factors enhancing the attractiveness of universities in competing for students and scholars (Kozłowski 2017; Sikorski et al. 2020).

## Findings

**Fig. 2:**
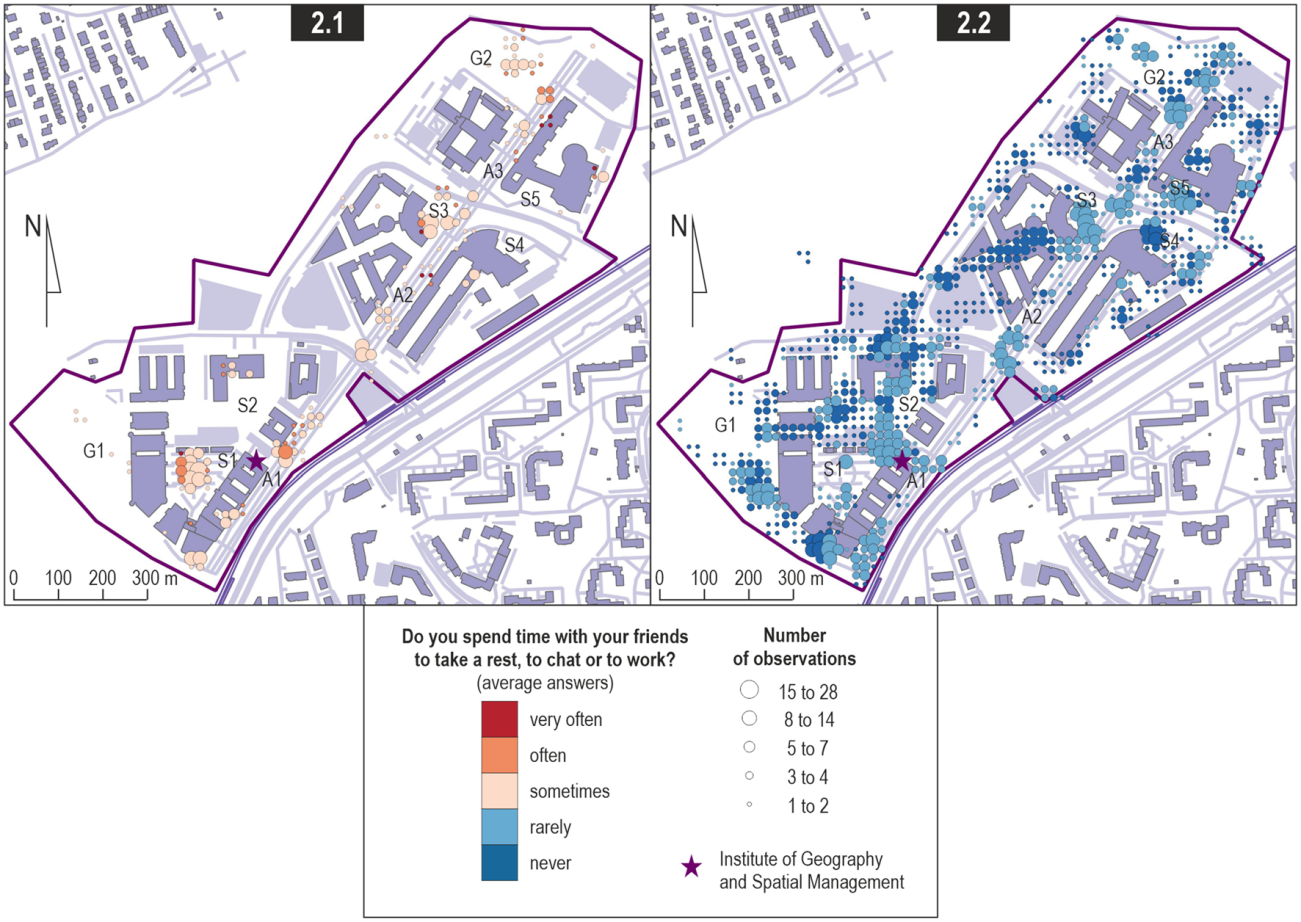
Locations where the crowdsensing study participants spent free time. *Source* Own study

The first key finding about the places where the students participating in the study spent their time (with friends, e.g. to rest, talk or work) for activities other than those related to studying itself, is that they explore the campus space poorly (Fig. 2, Map 2.2)—the size of their inactive space within the campus is considerable (see Yu et al. 2018). On account of its proximity, they occasionally stay within the inner courtyard (S1) of the complex where their institute is located as well as along the section of the pedestrian avenue (A1) that leads to the nearest tram stop. As for the remaining sections of the campus, they only go to one of the squares in front of another faculty (S3) and to green areas across the campus (G2) (Fig. 2, Map 2.1). This may provide evidence the attractiveness of these two areas compared to the rest of the campus, as well as poor interest in the campus area in general.

**Fig. 3:**
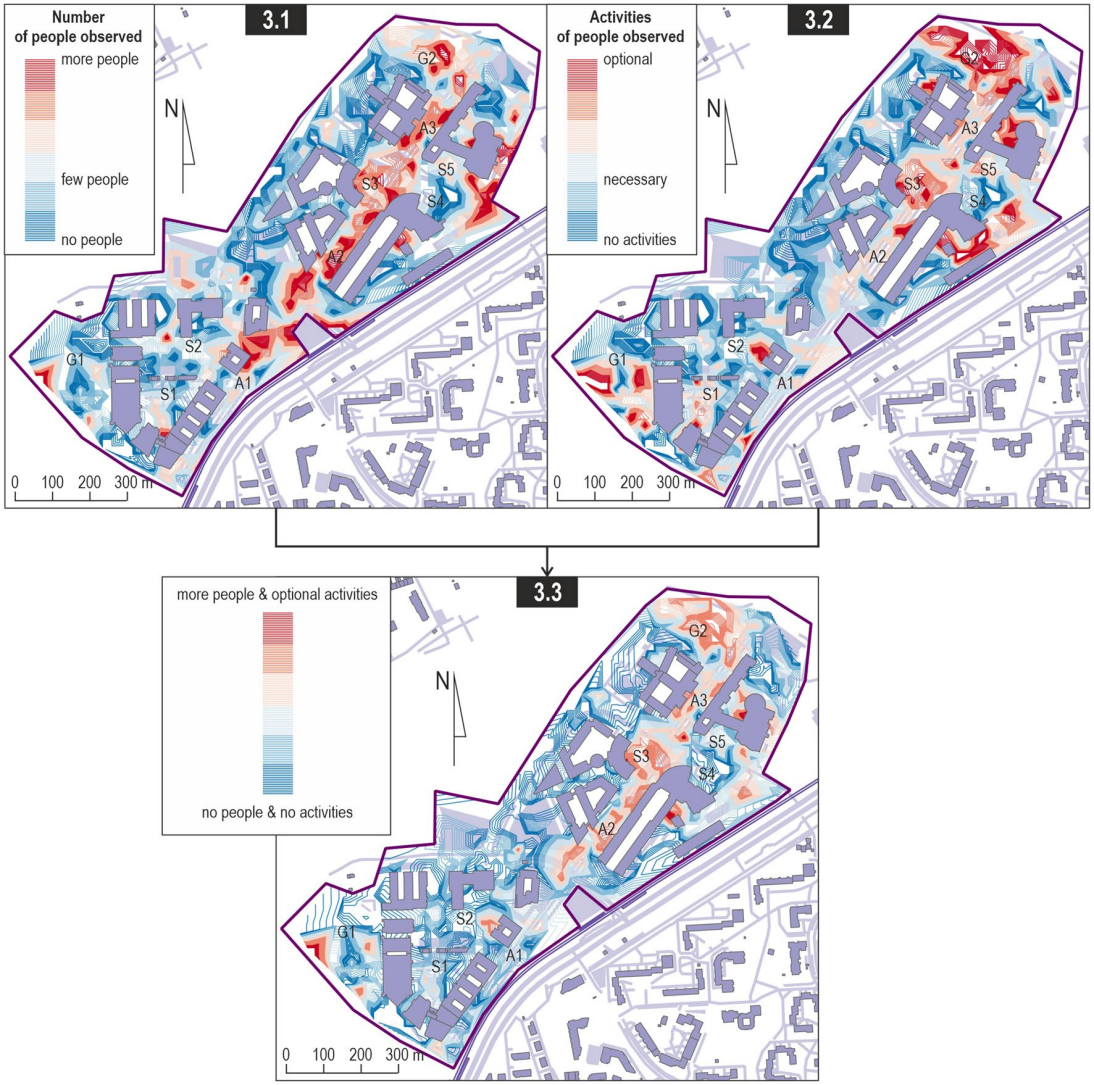
Presence and activity of people observed during the crowdsensing study. *Source* Own study

Observations of the behaviour of other campus users provide more in-depth information. A greater number of people were observed in the middle (A2) and northern (A3) sections of the pedestrian avenue (Fig. 3, Map 3.1). The streams of people moving towards the tram stops are also clearly visible. The only public space in which more users were observed was the square in front of one faculty (S3), with few people (S1 and S5) or almost no one (S2 and S4) in the other squares. With regard to green areas, more people stayed in the northern (G2) than in the southern (G1) part.

The behaviour patterns of campus users give a more nuanced picture of the campus space (Fig. 3, Map 3.2). The only spaces that prove their attractiveness, as evidenced by optional activities, are again the abovementioned square (S3) and the park in the northern part of the campus (G2). The main avenue has a mixed character along most of its course, with the walking function prevailing in the southern section (A1) and with a slightly more pronounced social function in the middle (A2) and northern (A3) sections.

The map that links the number of users with their behaviour reveals three public spaces that can be considered attractive from the perspective of the concept of the learning landscape (Fig. 3, Map 3.3): the square in front of one of the faculties (S3) with the adjacent section of the pedestrian avenue (A2) and the park at the northern end of the complex (G2). The remaining areas that should by definition play the role of public spaces do not fulfil that role in reality, which limits the potential of the campus of integrating various groups of users.

**Photo 1:**
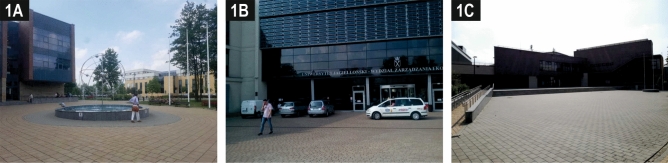
Three squares in front of main entrances to faculty buildings with the highest (S3, **A**), average (S5, **B**) and lowest (S4, **C**) attractiveness. *Source* Photos taken by the participants in the crowdsensing survey

**Photo 2:**
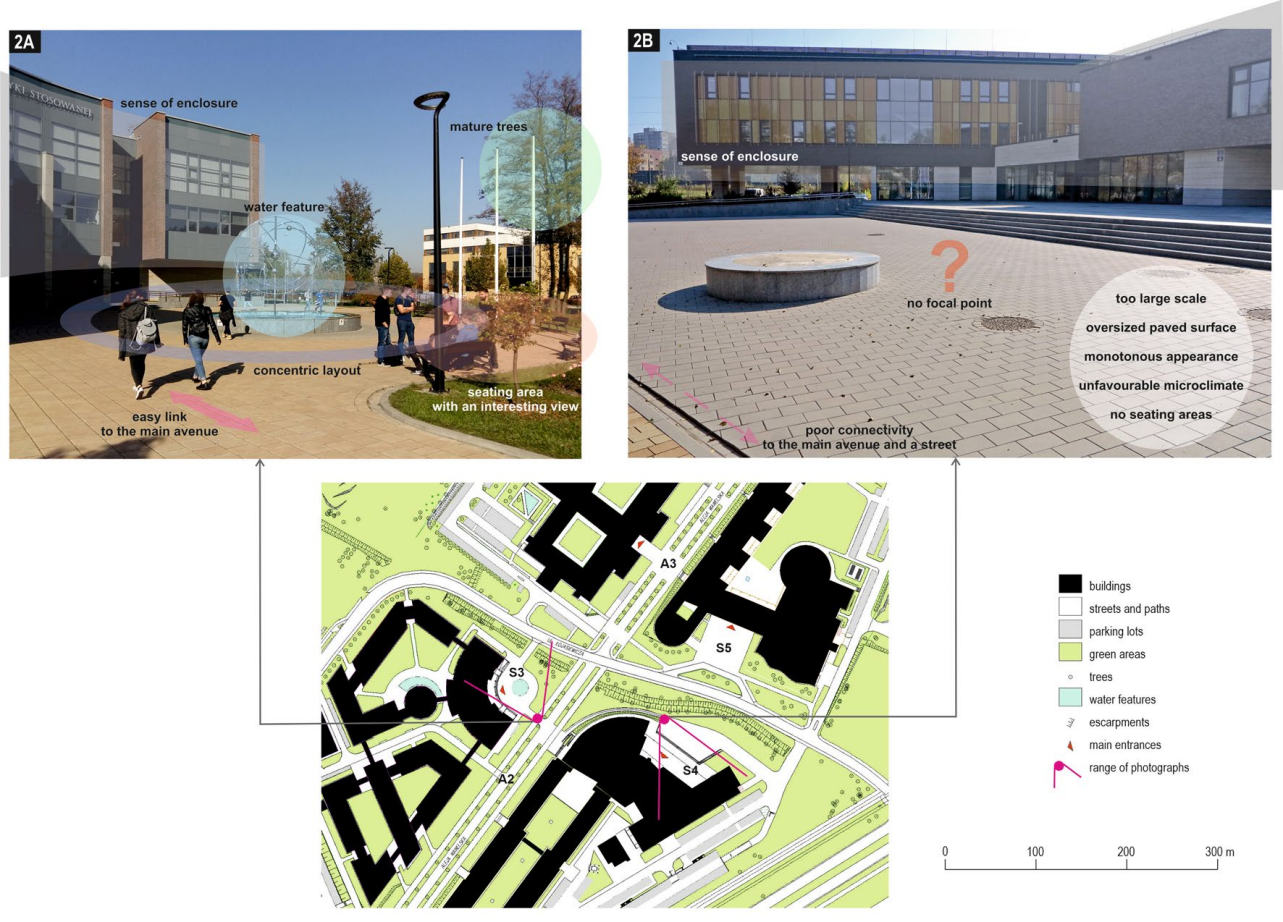
Expert assessment of the physical qualities of two public spaces, with the highest (S3, **A**) and the lowest attractiveness (S4, **B**): the former benefits from the presence of water, benches, trees, and shade, whereas the latter predominantly consists of paved surfaces. *Source* Photographs taken by authors in October 2018

The only attractive square (S3), out of the three located in front of the main entrances to faculty buildings (Photo 1), exhibits several positive features: the shading and the fountain give coolness on hot days and the benches arranged in a semicircle around it focus the activity of the users of this part of the campus. This square is a small ‘pocket’ of the pedestrian avenue and of the neighbouring street, which benefits from good visual and pedestrian connections, but in parallel, is characterised by a certain separateness and, unlike the monotonously arranged avenue, encourages pedestrians to stop here for a while. The concave entrance façade of the building, which faces the avenue, clearly marks the boundaries of the square. The neighbouring section of the pedestrian avenue gains attractiveness thanks to a dozen or so old trees which have been preserved here (Photo 2A).

The square in front of the neighbouring faculty building (S4) is paved and lacks trees and landscaping items, which means that the space is only used for passing from one place to another, playing a formal role, but not encouraging people to ‘hang-out’ there (Photos 1C, 2B). Another square (S5) (Photo 1B) displays an intermediate level of attractiveness. This square is located opposite the square discussed above (S4), but due to the street that separates them and the water ditch, the two squares are not connected either functionally or visually. Its shortcomings also include the lack of trees and, consequently, a lack of shade, which is important given the south-west exposure. This square’s primary role is to serve as the main, official entrance, despite passenger cars and delivery vans being parked there. In addition, owing to the lack of coordination between the location of the tram stops and the overall spatial layout of the campus, users are more likely to use the side entrance which is closer to the stops.

**Photo 3:**
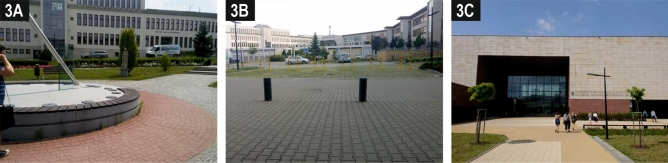
Despite the presence of the sundial and statues, square S1 (**A**) does not encourage people to spend their free time there due to the lack of shade and the feeling of being watched. Square S2 is divided by car parks (**B**), but the presence of the Nature Education Centre (**C**) means that there are better prospects for this part of the campus in the future. *Source* Photos taken by the participants in the crowdsensing survey

The other two public spaces (S1 and S2) are of a different nature. They are parts of a large courtyard in the southern, oldest (from 2000s) section of the campus, separated by a technical and utility building. The first area containing a distinctive sundial that formed the most eye-catching element for the participants (Photo 3A) is slightly more attractive. The courtyard, sloping towards the north, is devised as a green area with paved surfaces of access pathways and access roads. In the centre of the area, in addition to the sundial, there is a circle of monuments, benches and trees. The internal courtyard walls are plain and passive at the ground floor level with few windows and doors. The respondents appreciated the presence of greenery and benches but emphasised the lack of shade, which was not provided by the low trees planted there.^1^ This made the people present there feel as if they were being watched by people inside the buildings around. As a result, on average there were only a few people within the space, which was primarily used for passing between the wings of the complex. This is exacerbated by the lack of other functions that would stimulate those using this space. The poor connection between this area and the pedestrian avenue is another drawback.

The last of the squares (S2) is poorly connected with the adjacent courtyard (S1) and with the pedestrian avenue. Although it leads to the entrances to five buildings, which guarantees a large number of users, the spatial links between the buildings are chaotic, and the impression is exacerbated by the clumsy layout of vehicular and pedestrian routes. Those participating in the study drew attention to the car parks which dominate this space and which many pedestrians are forced to cross (Photo 3B). The only interesting accent for some of the respondents was the weather station located there; however, this does not encourage any activity. The presence of benches and greenery arranged in some parts of this square was also appreciated. Nevertheless, the results of the observations imply that it is almost entirely a zone that those on campus exclusively consider as an area they have to cross, being unable to find any reason to stop and socialise there. However, this space has huge as yet unexploited potential on account of the presence of the Nature Education Centre, which plans to organise events popularising science in the vicinity of the building in the future (Photo 3C).

**Photo 4:**
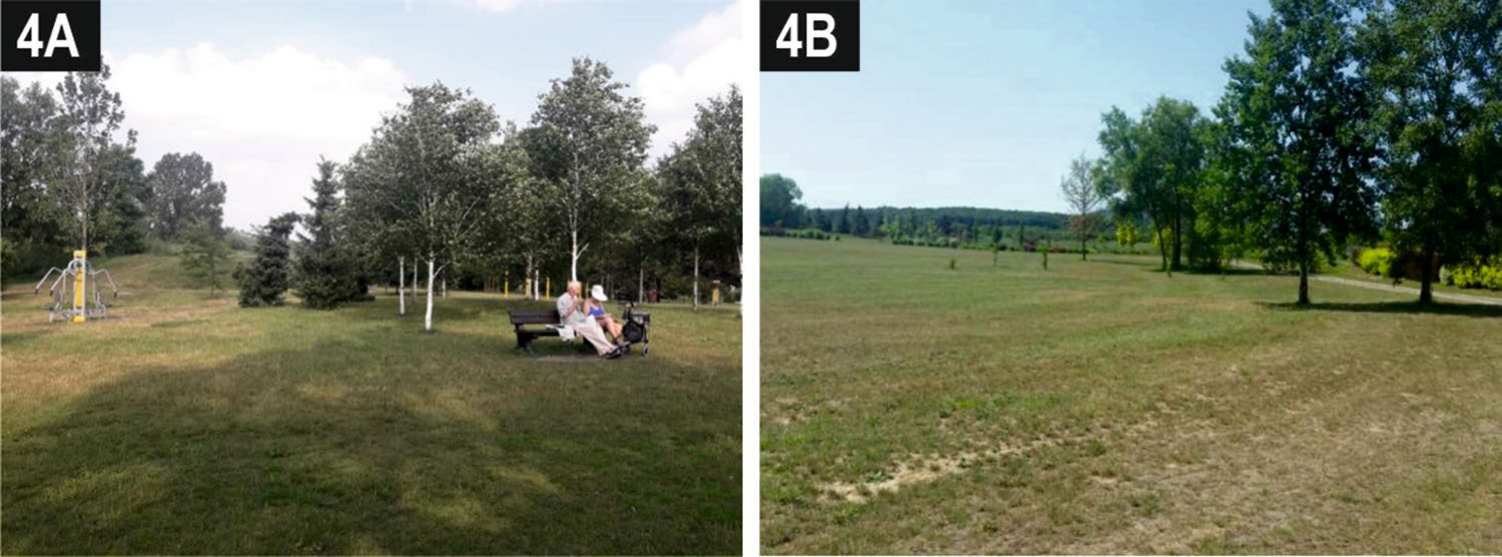
Park G2 (**A**) offers rest and active recreation opportunities to various groups of users. Park G1 (**B**) has untapped potential with regard to the integration of students and academics with the local community due to the lack of a suitable connection with other public spaces on the campus. *Source* Photos taken by the participants in the crowdsensing survey

The differences between the two existing green areas in the outermost parts of the campus have also been noted by respondents. Park G2 (Photo 4A), which is better connected with the avenue (A3), provides a place of rest both for the academic community and for the residents of neighbouring estates. The lower popularity of the other park area (G1) (Photo 4B), despite its natural value, stems from the fact that it is cut off from the other public spaces on the campus.

## Discussion and conclusions

The crowdsensing-based technique employed for collecting data on user behaviour yielded valuable insights. These findings are instrumental in understanding the functionality of campus public spaces and can aid in their improvement. The technique facilitated a large volume of observations, which, upon aggregation and visualization, can be compared with expert assessments of each site. Furthermore, the substantial amount of visual material provided by respondents offered additional support for these interpretations. Nevertheless, this approach does have limitations. Like other quantitative methods, it falls short in providing in-depth understanding of user motivations. Additionally, despite the seemingly attractive form and the apparent ease of use of the mobile application, we encountered difficulties in recruiting participants. This may be an evidence of low interest of campus users in the spaces where they study, work or—just like the residents of the nearby estates—spend their free time. It is advisable that future crowdsensing-based studies be expanded to include other categories of users and accompanied by additional quantitative and qualitative research methods (e.g. research walks).

Despite the above shortcomings, the results of the crowdsensing study and expert assessment confirmed that, at the time of the study, most of the analysed squares, courtyards, pedestrian paths and green areas analysed failed to fulfil the functions expected from modern public spaces (see Carmona 2015, 2019), thus weakening the role of the campus in the wider urban structure (Table 1). These spaces did not encourage people to stop and spend their time there, and were not conducive to planned or serendipitous encounters, since most of them failed to satisfy the key needs of their intended users (see Göçer et al. 2018), namely the needs for comfort, relaxation, commitment to activities, discovery, and entertainment. Two of the spaces, a square (S3) and a park (G2), stood out in a positive way. With regard to the square, this owed its appeal to several factors: the clearly defined walls, which gave it a distinctive character and a sense of enclosure while connecting it visually and physically with the avenue; the presence of water and shade; the inclusive, concentric arrangement of benches. In the case of the park, the attractiveness lied in the preserved old trees, the passive and active relaxation opportunities, and the unpretentious, varied layout, which is different from the highly ordered and monotonous design of the avenue. The former space (S3) may be conducive to socialising, while the latter (G2) to relaxation and recovery from mental fatigue (see Seitz et al. 2014).

**Table 1:** Assessment of the campus public spaces with recommendations for improvements

Public spaces	Use	Positive aspects	Negative aspects	Recommendations for improvements
Pedestrian avenue A1-A2-A3	Low (A1) to high (A2 and A3)	- Legibility and clarity of the urban composition, - Partly mixed character, social functions in the middle and northern sections	- Very plain, disconnected from the main entrances of most of the faculty buildings, - Lack of shade, only small formed trees, - Poor quality paving materials - Lack of outdoor activities	- Improve links with adjacent streets, public spaces and buildings and introduce nodes of activity while maintaining the continuity of the avenue, - Prioritise pedestrian avenue over the streets it is crossing by introducing traffic-calming measures, - Add more prominent endings of the avenue, - Diversify street furniture and its arrangement, - Use better-quality paving materials that comply with the principles of universal design, - Introduce native tree species with larger crowns, providing more shade (while keeping central viewing axis), and until then use shade sails as a temporary solution, - Remove some of the car parks adjacent to the avenue and transform them into green spaces, - Introduce outdoor food facilities, classrooms, exhibition spaces, etc
Square S1	Medium	- Presence of numerous landscaping elements and one recognisable symbolic feature (sundial)	- Lack of shade, lack of privacy - Unused potential of pavilions utilised for technical purposes only	- Ensure more privacy by planting native tree species with larger crowns, - Repurpose the auxiliary pavilions separating S1 and S2 e.g. for food establishments
Square S2	Low	- Some greenery and benches, - The Nature Education Centre (NEC) attracts visitors from outside the university	- Limited connectivity with S1 and the avenue A, - Conflicts of pedestrian and vehicular traffic, - Oversized paved surface in front of the NEC	- Separate pedestrian and vehicular traffic by reducing car-park size, - Redesign space in front of the NEC to create an extension of the indoor museum activities, e.g. outdoor event and exhibition space, waiting area and meeting point, - Provide more seating arrangements including L-shaped benches and benches facing each other, - Introduce shade in front of the NEC (larger trees, shade sails)
Square S3	High	- Offers shade from a few mature trees, has a water feature, bench layout fosters social contacts, - Spatially self-contained yet connected to the avenue A		- Use better-quality paving materials that comply with the principles of universal design
Square S4 and S5	Low (S4) to medium (S5)		- Dominated by large expanses of paving, - Disconnected from the avenue (A), - S4 lacks visibility, greenery, shade, and street furniture	- Reduce paved areas, increase green spaces, and provide shade, - Develop banks of the drainage ditch by strengthening its retention capacities, - Provide more diversified seating arrangements including L-shaped benches and benches facing each other, - Create a convenient pedestrian link between squares S4 and S5, - Eliminate illegal parking with use of street furniture and landscaping, - Introduce works of art symbolically connected with neighbouring faculties
Park G1	Low		- Strongly disconnected from S1 and A	- Create better and more visible connection with squares S1 and S2 and with the avenue A1 - Redesign landscaping by strengthening biodiversity and by underlining the natural character of the stream, - Introduce more diverse street furniture, - Provide more diversified seating arrangements including L-shaped benches and benches facing each other
Park G2	High	- Relatively attractive, varied, with larger native trees, with several levels, - Attracts people to spend time there, including at the outdoor gym	- Relatively good connectivity with the avenue A	- Strengthen its accessibility and visibility from the avenue A

The assessment of public spaces of the Third Campus of Jagiellonian University is influenced both by the large-scale axial layout and the specific solutions regarding the urban interiors and urban details. With the scenic qualities of the area taken as the starting point, the urban layout of the campus was founded on classical urban planning, with axes closed by prominent buildings. It expressed the desire to return to the ‘classical’ form of the city characteristic of the post-modern period. The coordination plan, with a powerful element of creativity, has provided the university area with an internal structure where the primary role is played by highly hierarchical public spaces. Unfortunately, the subordination of the spatial composition to axes oriented towards distant views at the expense of the most convenient (shortest) pedestrian routes (mainly to and from tram stops) means that the potential of the learning landscape of these underused spaces is not tapped (similar observations were made by Biddulph 1999).

The under-utilisation of public spaces is partly attributable to the functional programme decisions which resulted from the traditional silo mentality at universities (see Biddulph 1999; Winnicka-Jasłowska 2015): the construction of separate buildings for the individual faculties and institutes, their inward-oriented layout, the shortage of common university buildings and a small share of buildings with functions other than educational and scientific. The complex lacks dining establishments accessible from the outside which could activate the space. In addition, most of the other accompanying programmes are hidden inside the buildings, which limits their use by outsiders—the negative consequences for the businesses on the campus has become particularly strongly felt during the Covid-19 pandemic.

In our opinion, a closer look at how the campus space is arranged reveals a clear primacy of formality and monumentality over comfort and diversity, which is a consequence of misconceptions about what constitutes a successful university space. The campus space lacks diversity to the point of being monotonous. It does not ensure social comfort since it fails to offer the diversity that would allow people to choose their own place in space: in the sun or in the shade, in view or hidden, alone or in a group, etc. (see Gehl 2008; Whyte 2018; Carmona 2019). Despite the advantages resulting from it being hierarchical, the space between the buildings is, with a few exceptions, arranged in a repetitive manner with no care taken to exploit the existing variety, finished with mediocre materials and street furniture, and lacking sophisticated urban details. The main pedestrian artery forms a space subordinated to geometry, and its greenery is landscaped only to admire it, rather than to provide shelter and make informal use of it. It is far from the vision of a learning landscape that provides opportunities for networking, exchanging experiences, and experimenting.

The study indicated that, despite the vastness of the entire complex, the campus lacks solutions that add convenience for users, such as places for individual and group learning, eating outside, playing sports and organising social and cultural events. Within the campus, students and staff mostly rush between buildings and tram stops or parking lots. Although pedestrian users prevail on the campus, their needs are not prioritised. Access roads cross the main pedestrian route on the campus, and car parking areas occupy a large proportion of the area. In our opinion, all this hinders the process of building a sense of identity with the campus, which is made worse by the limited possibilities for co-deciding concerning the space for study and work.

The complex, as a whole, fails to play the role of a multifunctional city area, but is merely a grouping of specialised research and educational facilities, isolated and disconnected from one another, and in this respect it seems to be a late execution of twentieth-century modernist thinking. The shortcomings of the public spaces on campus, revealed both by the crowdsensing study and the expert assessment, inevitably lead to asking questions about what is the vision of the campus other than just a composed group of buildings and what tools can be used to make such a vision a reality. It is necessary to take actions to promote the constant adaptation of the campus space to current and future challenges regarding education and research, as well as the relations between the university and the city.

Paradoxically, despite the major spatial barriers between the campus and its surroundings, the university area, especially the pedestrian artery and green areas, are mainly frequented for recreational purposes, including during the pandemic, by the residents of the surrounding housing estates who struggle with even greater shortages of good-quality public space and networked green areas. However, in terms of their spatial and temporal patterns of use, the academic community and local residents seem to miss each other within the campus area. Managers of the campus space should pay attention to external users in a more conscious and open manner, which can be treated not only as a fulfilment of the university’s public mission, but also as an investment in the future by building a friendly image of the university.

There is a need for further joint reflection and efforts towards reviving the public spaces on the campus—both in terms of their physical appearance and socialising activity (see Biddulph 1999; Arefi and Triantafillou 2005). Based on the outcome of the study, we propose a number of specific recommendations for each square (Table 1) that could be a starting point for their redesign. However, we believe that the transformation of all of them should be guided by overarching ideas: of inclusiveness for various groups of users (from inside and outside the academia), of creativity stimulation (e.g. through art and cultural events, also in more informal, temporary, out-of-the-box spaces, through academic and educational outdoor programming), and of future-oriented, sustainable model solutions (e.g. shared multi-mode mobility spaces, blue-green infrastructure, community gardens). A campus cannot be treated as a ‘finished’ space, but instead should be open to urban experimentation and prototyping, e.g. through place-making activities (see Jones 2017; O’Rourke and Baldwin 2016; Whyte 2018). Therefore, it is necessary to conduct regular post-occupancy evaluations of the buildings and public spaces, develop strategies on how to integrate the campus internally and connect it with the city, and identify areas in most urgent need of intervention^2^ and design them in a participatory manner to engage the users and create bonds with the campus space. As a result of this, the campus spaces will be better able to fulfil the expected function of a learning landscape within the wider urban structure.

## Data Availability

The data presented in this study are available on request from the corresponding author.

## Footnotes

1 In 2019, in response, inter alia, to recommendations issued on the basis of the results of the present study, larger trees were planted.

2 Since the time of the study, campus space underwent minor changes. Based on the recommendations from the study, a competition was organised in 2019 for the development of one of the campus garden squares. It aimed on creating a recreational space respectful of local biodiversity. The winning project has been executed in 2020–2022. Moreover, in 2021 three new seating areas were introduced as a part of urban prototyping at the campus.
